# Current Trends in Fabrication of Biomaterials for Bone and Cartilage Regeneration: Materials Modifications and Biophysical Stimulations

**DOI:** 10.3390/ijms20020435

**Published:** 2019-01-20

**Authors:** Agata Przekora

**Affiliations:** Chair and Department of Biochemistry and Biotechnology, Medical University of Lublin, W. Chodzki 1 Street, 20-093 Lublin, Poland; agata.przekora@umlub.pl; Tel.: +48-81-448-7026

**Keywords:** metal ion substitution, antibacterial biomaterials, hydroxyapatite, atmospheric pressure plasma, magnetic field, low-intensity pulsed ultrasound, piezoelectric biomaterials

## Abstract

The aim of engineering of biomaterials is to fabricate implantable biocompatible scaffold that would accelerate regeneration of the tissue and ideally protect the wound against biodevice-related infections, which may cause prolonged inflammation and biomaterial failure. To obtain antimicrobial and highly biocompatible scaffolds promoting cell adhesion and growth, materials scientists are still searching for novel modifications of biomaterials. This review presents current trends in the field of engineering of biomaterials concerning application of various modifications and biophysical stimulation of scaffolds to obtain implants allowing for fast regeneration process of bone and cartilage as well as providing long-lasting antimicrobial protection at the site of injury. The article describes metal ion and plasma modifications of biomaterials as well as post-surgery external stimulations of implants with ultrasound and magnetic field, providing accelerated regeneration process. Finally, the review summarizes recent findings concerning the use of piezoelectric biomaterials in regenerative medicine.

## 1. Introduction: Biomaterials for Bone and Cartilage Regeneration 

The aim of the engineering of biomaterials is to fabricate a biocompatible scaffold that would support or ideally enhance regeneration of tissue after biomaterial implantation within the injured area. Optimal scaffold for bone and cartilage regeneration should have good surgical handiness and mechanical properties (especially Young’s modulus value) adjusted to the implantation area, e.g., Young’s modulus (*E*) value = 0.4–0.83 MPa for articular cartilage [1,2], *E* = 0.5–3 GPa for trabecular bone [3], and *E* = 15–23 GPa for cortical bone [4,5]. It should be noted that flexible biomaterials possessing lower stiffness (lower *E* value) compared to the host bone can be used only in non-load bearing implantation areas. These kinds of materials under exposure to the mechanical load may exert constant physical pressure to the surrounded bone tissue, resulting in excessive ossification. When material reveals superior stiffness compared to the host tissue at the implantation area, implant takes whole mechanical load and shields the bone cells from mechanical stimulus responsible for induction of bone formation process, causing excessive bone resorption and implant loosening [6,7]. Therefore, to achieve biomaterial showing mechanical properties close to natural bone, researchers primarily produce composite materials made of polymers—mimicking flexible organic part of the bone—and bioceramics, e.g., hydroxyapatite (HA) [8,9,10,11,12], α- and β-tricalcium phosphate (TCP) [13,14], low-temperature calcium phosphate cements (CPCs) [15,16,17,18], or bioactive glass (BG) [19,20], which imitate natural mineral of the bone (HA), providing better mechanical properties of the resultant scaffold. Biomaterials for cartilage regeneration should be more elastic compared to the bone scaffolds and are mainly produced using biocompatible polymers (natural and synthetic) often resembling those occurring in natural cartilage tissue, e.g. chitosan, hyaluronic acid, collagen, fibrin, silk, alginate, polylactic acid (PLA), poly(3-caprolactone) (PCL), or poly(l-lactide-co-caprolactone) (PLCL) [21,22]. To obtain better mechanical and biological properties, cartilage scaffolds are also produced as composite materials, using combination of different polymers, e.g., agarose and poly(ethyleneglycol) (PEG) [23], collagen and PEG [24], or chitosan and hyaluronic acid [25].

Biomaterials for regenerative medicine applications should be favorable to cell adhesion, proliferation and differentiation to ensure rapid regeneration process at the site of injury. To obtain highly biocompatible scaffolds promoting cell adhesion and growth, materials scientists are still searching for novel modifications of biomaterials to obtain higher surface roughness, wettability, and surface free energy, allowing for better adsorption of cell adhesive proteins (e.g., laminin, fibronectin, vitronectin) and thereby more effective cell attachment [26,27,28,29,30]. Furthermore, to obtain better regeneration rate, scientists reach for such a solution like external physical stimulation of biomaterial after its implantation with low-intensity pulsed ultrasound (LIPUS) [31], magnetic field [32], or electrical forces [33]. Ideally, biomaterials for bone and cartilage regeneration should not only promote new tissue formation at the site of injury, but also protect the wound against biodevices-related infections, which may cause prolonged inflammation and biomaterial failure [34]. Thus, there is a great tendency in engineering of biomaterials to produce implants with antibacterial activity against Gram-positive and Gram-negative bacteria which are typical of infections related to orthopedic surgery: *Staphylococcus aureus*, *Staphylococcus epidermidis*, *Escherichia coli*, and *Pseudomonas aeruginosa* [34,35,36].

This review presents current trends in the field of engineering of biomaterials concerning application of various modifications and biophysical stimulation of biomaterials to obtain implants allowing for fast regeneration process and providing long-lasting antimicrobial protection at the site of injury.

## 2. Biomaterial Modifications with Metal Ions 

### 2.1. Biomaterials with Osteopromotive Properties

Researchers have recently focused on the modifications of biomaterials with various metal ions in order to give them better biological properties. Biomaterials are usually modified by ionic substitution of bioceramics (HA and BG) or by incorporation/deposition of metal ions on the surface of the metal implants. Since the main goal of engineering of biomaterials and tissue engineering is enhancement of regeneration process at the site of implantation, there is a growing trend towards modification of implantable materials with metal ions, which provide osteopromotive properties (Table 1), e.g., magnesium (Mg^2+^), zinc (Zn^2+^), copper (Cu^2+^), strontium (Sr^2+^), cobalt (Co^2+^), lithium (Li^+^), and fluoride (F^−^) [37]. Galli et al. [38] conducted physical deposition of Mg ions within mesoporous titanium (Ti) films coated on titanium threaded screws and demonstrated that local release of Mg ions significantly improved implant osseointegration with tibia of rabbits. Liu et al. [39] produced biodegradable Mg–Cu alloys that thanks to constant Cu^2+^ and Mg^2+^ release were proved to enhance bone formation (in vitro mouse calvarial preosteoblasts model—MC3T3-E1 cell line) and angiogenesis (in vitro model of human umbilical vein endothelial cells—HUVEC cell line) and also to possess long-lasting antibacterial properties (studies on *S. aureus*). Yusa et al. [40,41] modified titanium surfaces with zinc to obtain biomaterials with large potential in orthopedic applications. The release of Zn ions from Zn-modified titanium implant promoted osteogenic differentiation leading to prominent bone formation process (studies on in vitro cellular models: human bone marrow-derived mesenchymal stem cells—BMDSCs and human dental pulp stem cells—DPSCs). Similarly, Thian et al. [42] synthesized Zn-doped HA and demonstrated enhanced proliferation and differentiation potential of human adipose tissue-derived mesenchymal stem cells (ADSCs) on the biomaterial made of Zn-doped HA. In turn, Andersen et al. [43] showed that Sr-modified surface of titanium implant improved osteogenic differentiation of human DPSCs and osseointegration of implant with femur bone of rats. Fluoride ions are also known to promote osteogenic differentiation and thus they are often used for HA substitution [44]. Uysal et al. [45] synthesized HA co-doped with Zn^2+^ and F^–^ ions and showed that modified HA not only possessed better mechanical properties compared to pure HA, but also increased proliferation and bone alkaline phosphatase activity (bALP) of osteosarcoma derived osteoblast-like cells (Saos-2 cell line). Ionic substitution is also often applied for BGs. Khorami et al. [46] used Li^+^ for substitution of 45S5 BG and proved that modified biomaterial provided higher proliferation rate and bALP activity of primary rat calvarial osteoblasts. Whereas Wu et al. [47,48] produced Co- and Cu-containing mesoporous BGs, which were demonstrated to promote proliferation and osteogenic differentiation of human BMDSCs. 

### 2.2. Biomaterials with Antimicrobial Properties

Because implant-related infections are a severe problem in orthopedic surgery, more often researchers put emphasis on the modification of biomaterials with metal ions revealing antimicrobial properties, such as: Zn^2+^, Cu^2+^, and predominantly silver (Ag^+^) which is commonly known for its broad spectrum of antimicrobial activity against bacteria and fungi (Table 2). Nevertheless modification of biomaterials with antimicrobial metal ions has some limitations since high concentrations of Ag^+^, Zn^2+^, and Cu^2+^ showing satisfactory antimicrobial action may also be lethal to the eukaryotic cells.

**Figure 1:**
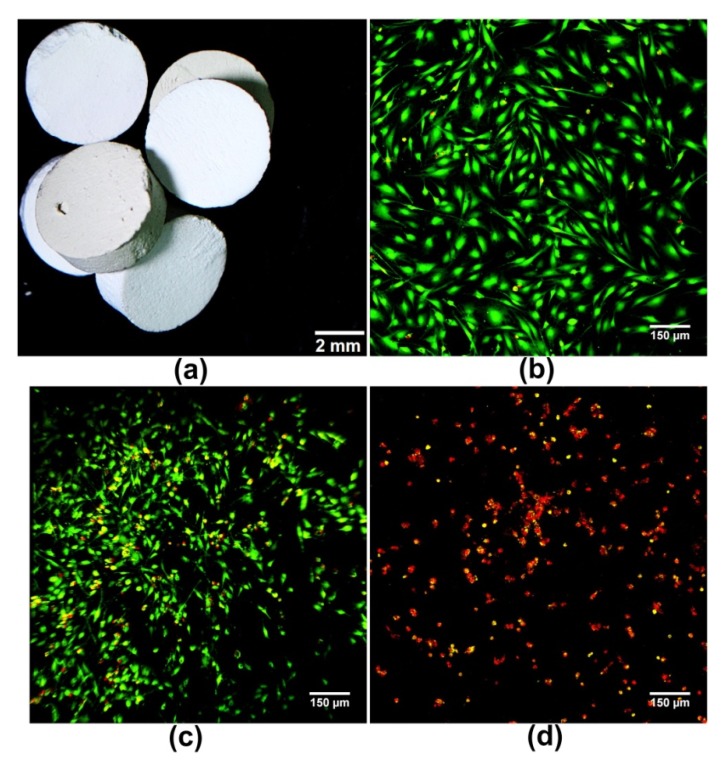
Cytotoxicity evaluation of antibacterial bone cements containing Ag-doped hydroxyapatite (HA): (**a**) shows fabricated bone cements; (**b**) shows control healthy MC3T3-E1 preosteoblasts cultured on polystyrene after live/dead staining; (**c**) and (**d**) show live/dead staining of MC3T3-E1 cells with reduced viability grown directly on the antibacterial bone cements with low (**c**) and high (**d**) concentration of Ag-doped HA [65] (green fluorescence—viable cells stained with calcein-AM, red fluorescence—nuclei of dead cells stained with propidium iodide).

Rau et al. [64] fabricated calcium phosphate bone cements containing Ag-doped TCP revealing the inhibitory effect towards pathogenic *E. coli*, but the paper does not present cytotoxicity studies on eukaryotic cells. Whereas in our recent studies [65] we produced α-TCP-based bone cement containing CaCO_3_ and Ag-doped HA showing antibacterial effect against *S. aureus*, *S. epidermidis*, and *E. coli*. However, produced bone cements revealed also cytotoxic effect against eukaryotic cells (MC3T3-E1) (Figure 1). In turn, Lim et al. [66] produced Ag-doped HA and demonstrated its high antibacterial activity against *S. aureus* and non-toxicity against human BMDSCs. Other metal ions that are commonly used for antimicrobial modifications of biomaterials include Zn^2+^ and Cu^2+^. Thian et al. [42] produced Zn-doped HA that significantly reduced growth of *S. aureus* bacteria on its surface and showed non-toxicity against human ADSCs. While Wu et al. [48] used Cu^2+^ for substitution of BG and obtained biocompatible (studies on human BMDSCs) porous biomaterial with high antibacterial activity against *E. coli.* Interestingly, apart from typical antimicrobial metal ions, also lithium (Li^+^) [63] and cerium (Ce^3+^) [62,67] have been reported to possess potential to be used as an effective antimicrobial agent for biomaterial modification.

## 3. Plasma-Modified Biomaterials 

### 3.1. Biomaterials with Improved Biocompatibility

Plasma treatment may be used for surface functionalization with hydrophilic chemical groups and for improvement of surface properties of biomaterials like wettability, roughness or surface free energy. All mentioned features have great impact on cell adhesion and thereby material biocompatibility. The effectiveness of plasma modification highly depends on substrate gas used for the treatment, reactor design, or the type of biomaterial subjected to the modification. In engineering of biomaterials, plasma (recently special attention has been paid to atmospheric pressure plasma) combined with argon, oxygen, air, amonia, or nitrogen gas is most often used for surface modifications of primarily polymeric materials [68,69,70,71]. Kostov et al. [72] modified polyethyleneterephthalate, polyethylene (PE), and polypropylene with cold atmospheric plasma jet using argon gas and obtained polymers with increased roughness and wettability. Sagbas et al. [71] used argon/oxygen atmospheric plasma to increase wettability and wear resistance of ultra-high molecular weight PE. They proved that surface treatment with argon plasma followed by oxygen plasma led to incorporation of polar groups (mainly hydroxyl groups), which are known to promote cell adhesion. Jordá-Vilaplana et al. [73] increased surface free energy as well as improved wettability and roughness of PLA material by its modification with air atmospheric plasma. Griffin [70] et al. compared the effect of argon, oxygen, and nitrogen plasma treatment on biocompatibility of polyurethane (PU) scaffold. They demonstrated that among all tested plasma treatments, oxygen plasma had the greatest effect on surface properties of the scaffold. The oxygen plasma significantly decreased water contact angle (increased wettability) and meaningfully increased roughness of the material. Nevertheless, argon-modified material not only revealed the highest protein adsorption ability and human dermal fibroblast adhesion effectiveness, but also the highest tissue integration and angiogenesis after scaffold subcutaneous implantation in a mouse model, compared to oxygen and nitrogen-plasma treated scaffolds. In another study, Griffin et al. [74] used oxygen plasma for functionalization of PU-based scaffold with amino (NH_2_) groups (plasma polimerisation with the use of allylamine) and carboxyl (COOH) groups (plasma polymerisation with the use of acrylic acid monomers). They showed that plasma functionalization of the biomaterials significantly improved their biocompatibility since COOH-functionalized material promoted chondrogenic differentiation, whereas NH_2_-functionalized scaffold enhanced osteogenic differentiation of human ADSCs. Chen et al. [69] used argon and nitrogen plasma for improvement of biocompatibility of polyurethane methacrylate (PUMA) and off-stoichiometry thiol-ene (OSTE-80) polymers. Plasma treatment introduced oxygen and nitrogen moieties on the surface of polymers, increasing surface energy, hydrophilicity, and chemical functionalities. As a consequence, biomaterials exhibited ability to promote HUVEC adhesion and proliferation. In turn, Wang et al. [75] applied helium cold atmospheric plasma for modification of PLA scaffold and they observed that plasma-treated polymer had significantly decreased water contact angle (from 70 ± 2° to 24 ± 2°) and drastically increased nano-scale roughness, resulting in improved biocompatibility (enhanced attachment and proliferation of primary human osteoblast and human BMDSC). 

### 3.2. Biomaterials with Antibacterial Properties

Plasma treatment is widely used not only for improvement of biocompatibility of biomaterials, but also to provide them antimicrobial properties. The antibacterial properties of medical materials may be achieved by immobilization of organic active reagents or introducing metal ions (Zn, Cu, Ag) into the surface as well as by modifications of surface morphologies (topography) and chemical composition (e.g., by introducing specific functional groups) to prevent attachment and proliferation of pathogenic bacteria [76,77]. To produce antibacterial films on the surface of biomaterials, some plasma-assisted modification techniques have been recently more often used, e.g. metal or gas plasma immersion ion implantation (PIII) or magnetron sputtering [76]. Zhang et al. [78] applied PIII to incorporate Ag into PE and obtained biocompatible (studies on normal human fetal osteoblast cell line—hFOB 1.19) and antibacterial surface (studies on *E. coli*). They also demonstrated that application of Ag PIII in combination with nitrogen PIII provided prolonged antibacterial activity; however nitrogen-containing functional groups (such as C–N and C=N) formed on the surface of PE reduced proliferation of hFOB 1.19 osteoblasts. In other studies by Zhang et al. [79,80], PE and medical poly(vinyl chloride) samples were modified using oxygen plasma, then precoated with antibacterial agents (triclosan or bronpol), followed by argon PIII to ensure that active agents strongly bonded to the surface of materials. Modified PE and poly(vinyl chloride) samples showed excellence antibacterial activity against *E. coli* and *S. aureus*, however the papers do not present biocompatibility test on eukaryotic cells. Whereas Wang et al. [81] applied nitrogen PIII for the production of biocompatible (studies on primary rat calvarial osteoblasts) poly(butylene succinate) samples with antibacterial activity against *E. coli* and *S. aureus*. 

## 4. External Biophysical Stimulation of the Biomaterials

### 4.1. Low-Intensity Pulsed Ultrasound Stimulation

Low-intensity pulsed ultrasound (LIPUS) is an external stimulation, which according to basic and clinical studies has ability to improve bone and cartilage healing process by promoting proliferation and differentiation of mesenchymal stem cells (MSCs) [82,83,84,85]. Moonga et al. [86] proved that LIPUS treatment reduced cell proliferation but significantly enhanced ECM mineralization of MC3T3-E1 preosteoblasts cultured directly on bovine trabecular bone scaffold. Carina et al. [31] demonstrated that 20-minute exposure of Mg-HA/collagen scaffold pre-seeded with human MSCs to the LIPUS improved colonization of biomaterial and enhanced osteogenic differentiation of stem cells. Zhou et al. [87] determined optimal LIPUS parameters for bone regeneration process (1.5 MHz, 20% duty cycle with 150 mW/cm^2^ intensity) and revealed that PE-based 3D printed scaffold containing arginine-glycine-aspartic acid-serene (RGDS) peptide and nanocrystalline HA under LIPUS stimulation greatly promoted proliferation and osteogenic differentiation of BMDSCs. In turn, Aliabouzar et al. [88] investigated the effect of LIPUS treatment on proliferation and chondrogenic differentiation of human MSCs seeded onto 3D printed PEG- diacrylate scaffold. They demonstrated that LIPUS stimulation (optimal parameters were found to be 1.5 MHz, 20% duty cycle with 100 mW/cm^2^ intensity) significantly increased MSC proliferation as well as enhanced glycosaminoglycan (GAG) and type II collagen synthesis. In another study, Aliabouzar et al. [89] used lipid-coated microbubbles combined with LIPUS to enhance proliferation and chondrogenic differentiation of human MSCs seeded onto 3D printed PEG-diacrylate hydrogel scaffold. They proved that application of LIPUS along with microbubbles significantly increased MSC proliferation up to 40% (up to 18% with LIPUS alone), promoted GAG synthesis by 17% (by 5% with LIPUS alone), and enhanced type II collagen production by 78% (by 44% with LIPUS alone).

### 4.2. Magnetic Field Stimulation

**Figure 2:**
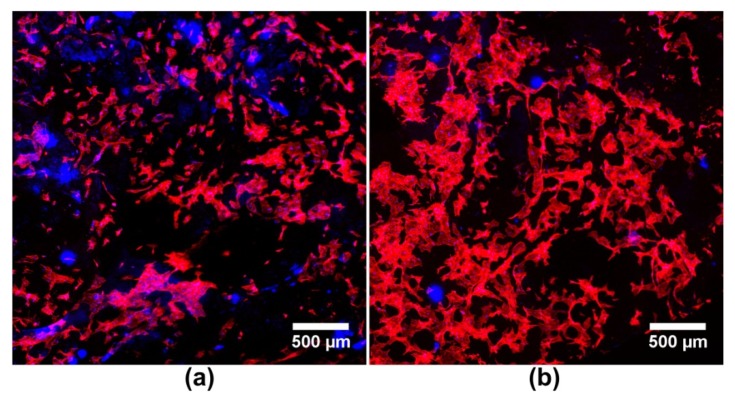
Confocal laser scanning microscope images of MC3T3-E1 preosteoblasts cultured on the surface of biomaterial without and with ferromagnetic nanoparticles (NPs): (**a**) shows MC3T3-E1 cells on chitosan-based scaffold without ferromagnetic NPs; (**b**) shows greater number of better spread MC3T3-E1 preosteoblasts on chitosan-based scaffold with ferromagnetic NPs incorporated within polysaccharide matrix (cell cytoskeleton was stained with AlexaFluor635phalloidin).

Magnetic field is another type of external biophysical stimulation proved to substantially improve regeneration process [32,90]. Therefore there is a trend in engineering of biomaterials towards production of biomaterials with magnetic nanoparticles (MNPs) incorporated [91,92]. Novel approach to bone and cartilage regeneration includes the use of MNPs-loaded scaffolds combined with magnetic field stimulation in order to accelerate tissue regeneration and in the case of bone implants also angiogenesis process. Samal et al. [93] fabricated MNPs-loaded silk fibroin scaffold, which exhibited outstanding hyperthermia properties under exposure to magnetic field and revealed capability to enhance adhesion and colonization of MC3T3-E1 preosteoblasts. Cai et al. [94] produced magnetic scaffold made of poly(l-lactide) (PLLA) nanofibers loaded with ferromagnetic (Fe_3_O_4_) nanoparticles (NPs), which was demonstrated to promote proliferation and osteogenic differentiation of MC3T3-E1 cells not only after stimulation with static magnetic field, but also in the absence of external stimulation. Interestingly, in our recent studies it was observed that the incorporation of ferromagnetic NPs within the scaffold itself (without external stimulation with magnetic field) may be sufficient to improve biocompatibility of the resultant biomaterial since MC3T3-E1 preosteoblasts cultured on the surface of magnetic chitosan-based scaffold exhibited better spreading and faster proliferation compared to the cells grown on the scaffold without ferromagnetic NPs (Figure 2). In turn, Yun et al. [95] showed enhanced osteogenic differentiation of primary mouse osteoblasts seeded onto magnetic MNPs-loaded PCL scaffold upon exposure to static magnetic field. Moreover magnetic field stimulation of MNPs/PCL scaffold implanted in mouse calvarium defects resulted in significantly improved bone regeneration (as demonstrated by the histological and microcomputed tomography analyses). Yan et al. [96] fabricated novel biocompatible (studies on human MG-63 osteosarcoma-derived cell line) magnetic scaffold by incorporating a spherical core-shell nano-iron oxide-hydroxyapatite (Fe_3_O_4_-HA) into PU. Whereas Heidari et al. [97] developed biocompatible (studies on human MSCs) chitosan/HA/nano magnetite (nano-Fe_3_O_4_) composite with improved mechanical properties. D’Amora et al. [98] produced magnetic scaffolds by incorporating Fe-HA NPs into a PCL matrix and demonstrated that magnetic stimulation led to improved proliferation of human MSCs, which were also better spread compared to the cells grown on the surface of unstimulated scaffolds. Aliramaji et al. [99] developed magnetic silk fibroin/chitosan/Fe_2_O_3_ scaffold and proved its great biocompatibility under a static magnetic field. While Anjaneyulu et al. [100] demonstrated that magnetic HA/Fe_3_O_4_ coatings on the Ti-6Al-4V material significantly improved bioactivity of the resultant biomaterial by developing Fe-OH groups on the surface. 

## 5. Piezoelectric Biomaterials

Piezoelectric biomaterials possess the ability to generate a bioelectrical signal upon exposure to mechanical stress (without an external power source), which subsequently promotes tissue regeneration at the site of implantation [101]. Since piezoelectric scaffolds resemble sensitive mechano-electrical transducers, they are usually applied to the implantation areas, which are exposed to mechanical loads [101,102]. Nevertheless apart from physiological mechanical load, piezoelectric implants may be also subjected to ultrasound [103] or electrical stimulation [102,104]. The piezoelectric biomaterials produce electric signal, which is similar to that occurring in natural extracellular matrix (ECM) during remodeling process of cartilage and bone tissue. In physiological environment, the compressive force on collagen fibers within ECM leads to the re-organization of dipole moment, resulting in negative charges. As a consequence, electrical signal reaches the cell membrane, leading to the opening of voltage-gated calcium channels. Increased intracellular calcium concentration results in activation of calmodulin, followed by the activation of calcineurin, which causes dephosphorylation of Nuclear Factor of Activated Cells (NF-AT) and its translocation into the nucleus, where along with other transcription factors NF-AT regulates expression of some growth factors, inter alia Transforming Growth Factor-β (TGF-β) and Bone Morphogenetic Proteins (BMPs), which are known to play crucial role in the formation of cartilage and bone [103].

Since the discovery of piezoelectricity in natural cartilage and bone, many piezoelectric biomaterials for regenerative medicine applications have been developed (Table 3). Chen et al. [105] fabricated biocompatible potassium sodium niobate (KNN) ceramics with piezoelectric constant of ~93 pC/N, which promoted growth of MC3T3-E1 preosteoblasts and revealed better protein adsorption capacity compared to non-polarized ceramics. Lv et al. [106] developed 0–3-type niobate-based lead-free composite ceramics with ZnO inclusions showing excellent piezoelectric properties and superior temperature stability. Tang et al. [107] produced HA/barium titanate (BaTiO_3_) composite material with piezoelectric constant of 1.3 pC/N to 6.8 pC/N dependent on the BaTiO_3_ content, which promoted osteoblast growth. Augustine et al. [108] modified widely known piezoelectric polymer-poly (vinylidene fluoride-trifluoroethylene) (PVDF-TrFE) by incorporation of zinc oxide (ZnO) nanoparticles and demonstrated that human MSCs and HUVECs cultured on the resultant scaffold showed higher cell viability, enhanced adhesion and proliferation compared to the cells grown on PVDF-TrFE material without ZnO. Damaraju et al. [109] investigated the effect of 3D fibrous PVDF-TrFE scaffold on osteogenic differentiation of human MSCs. They proved that piezoelectric scaffold exhibiting low voltage output promoted chondrogenic differentiation of MSC, whereas PVDF-TrFE scaffold with a high voltage output induced osteogenic differentiation of stem cells. Moreover, they revealed that electromechanical stimulus was more effective in promotion of cell differentiation compared to mechanical load. 

## 6. Concluding Remarks

Modern strategy to bone and cartilage regeneration includes the use of biomaterials to support new tissue formation and to accelerate healing process at the implantation area. However, biomaterials (especially made of natural biopolymers and containing active biomolecules) may induce biomaterial-related immune response. It should be noted that prolonged inflammation may result in oxidative damage of the implant and its loosening. Moreover, the surgery of biomaterial implantation is an invasive procedure, which is always associated with the injury of the tissue, carrying a risk of post-surgery infections. Therefore, scientists with the expertise in the field of engineering of biomaterials are still searching for new modifications of biomaterials to improve their biocompatibility, provide accelerated regeneration process as well as antimicrobial protection at the implantation site. To achieve this goal, biomaterials are often modified with the use of active biomolecules (e.g., growth factors, cytokines, Arg-Gly-Asp (RGD) sequences, antibiotics) or metal ions revealing antimicrobial or osteopromotive properties. However, implantable materials made of natural biopolymers or containing biological components (e.g., cells or cellular products) carry a risk of elicitation of immunogenic immune response, which is induced by dendritic cells followed by activation of lymphocytes [119]. Furthermore, clinical application of biomaterials containing active biomolecules may disturb physiological balance between growth factors and cytokines naturally occurring in a living organism. Regeneration of bone and cartilage tissue is controlled by local release of a number of biological molecules (growth factors and cytokines) followed by crosstalk between different cell types, inter alia osteoblasts, osteoclasts, macrophages, stem cells, neutrophils, and natural killers [120,121,122,123]. Therefore, the changes in local concentrations of some cytokines/growth factors caused by modified with biomolecules material may exert negative effect on subsequent regeneration process after implantation. Thus, there is a clear need to understand the kind of the modes of action of biomolecules used for biomaterial modification in order to predict potential effect of modified implant on response of immune and precursor cells [124]. In this context, post-surgery biophysical stimulation of regeneration process appears to be safer solution in modern regenerative medicine. Consequently, magnetic field, ultrasound or electrical forces are more often applied to provide better healing process after biomaterial implantation. Taking into account great technological development, it is very likely that in the nearest future, routine treatment of cartilage and bone defects will involve implantation of smart antimicrobial biomaterial and remote stimulation of healing process by the patient at home by simply wearing a special orthopedic brace generating e.g. LIPUS.

## Figures and Tables

**Table 1:** Examples of osteopromotive biomaterials produced by modification with metal ions.

Metal Ion	Type of Biomaterial	Experimental Model	Biomaterial Effect on Bone Regeneration	Ref.
Mg^2+^	Ti threaded screws with Mg-incorporated mesoporous TiO_2_ coating	In vivo rabbit model	Improved biomaterial osteoconductivity, enhanced expression of genes related to bone regeneration process	[38]
Mg^2+^ Sr^2+^	Pure Ti samples with Mg or Sr ions deposited on the surface	In vivo rabbit model; In vitro model: MC3T3-E1 cell line	Enhanced proliferation and osteogenic differentiation in vitro, improved biomechanical strength and osseointegration in vivo	[49]
Mg^2+^ Zn^2+^ Sr^2+^	Ti implants with Mg- or Zn- or Sr-doped HA coating	In vivo rat model	Increased new bone formation, improved implant osseointegration	[50]
Zn^2+^	Zn-modified Ti sponge	In vitro model: human DPSCs	Increased osteogenic differentiation and mineralization	[51]
Zn^2+^	Ti rods/plates with Zn-incorporated TiO_2_ coating	In vivo rat model; In vitro model: rat BMDSCs	Enhanced expression of genes related to bone regeneration process, improved bone formation process in vitro and in vivo	[52]
Sr^2+^	Ti implant with Ti-Sr-O coating	In vivo rabbit model	Improved early implant osseointegration	[53]
Co^2+^	Alginate/collagen/α-TCP scaffold with Co incorporated	In vitro model: rat BMDSCs	Enhanced angiogenic properties of cells and osteogenic differentiation	[54]
Sr^2+^ Co^2+^	BG co-substituted with Sr and Co	In vivo rabbit model; In vitro model: human umbilical cord perivascular cells (HUCPVCs), Saos-2 cell line	Increased expression of genes related to osteogenesis and angiogenesis processes in Saos-2 and HUCPVC cells, respectively, improved bone healing process in vivo	[55]

**Table 2:** Examples of antimicrobial biomaterials produced by modification with metal ions.

Metal Ion	Type of Biomaterial	Demonstrated Antimicrobial Activity (Microbial Strain)	Effect on Eukaryotic Cells	Ref.
Ag^+^	Mesoporous 58S BG containing Ag	*Escherichia coli; Staphylococcus aureus*	Biocompatible (studies on primary rat calvarial osteoblasts)	[56]
Ag^+^ Zn^2+^	Ti-6Al-4V alloy with Ag- or Zn-doped HA coating	*Streptococcus mutans*	Reduced cell attachment (studies on human gingival fibroblast cell line—HGF-1)	[57]
Ag^+^ Cu^2+^	Ag- or Cu-doped HA/α-TCP Ag- or Cu-doped HA	*Staphylococcus aureus; Escherichia coli; Pseudomonas aeruginosa; Candida albicans*	Non-toxic, slightly reduced cell proliferation rate (studies on human lung fibroblast cell line—MRC-5)	[58]
Zn^2+^ Cu^2+^	Zn- or Cu-doped nanoHA	*Staphylococcus aureus; Escherichia coli*; *Candida albicans*	Not tested	[59]
Cu^2+^ Mg^2+^	Mg-Cu alloy	*Staphylococcus aureus*	Biocompatible (studies on MC3T3-E1 and HUVEC lines)	[39]
Cu^2+^	Chitosan biomaterial with Cu incorporated	*Escherichia coli; Staphylococcus carnosus*	Non-toxic at low Cu concentrations, toxic at high Cu concentrations (studies on mouse embryonic fibroblast cell line—MEF)	[60]
Cu^2+^	Mesoporous BG containing Cu	*Staphylococcus epidermidis; Staphylococcus aureus; Escherichia coli*	Not tested	[61]
Ce^3+^	Ce-doped nanoHA	*Escherichia coli; Staphylococcus aureus*	Not tested	[62]
Li^+^	Li-doped 58S BG	*Staphylococcus aureus*	Biocompatible (studies on MC3T3-E1 cell line)	[63]

**Table 3:** Examples of piezoelectric biomaterials and their effect on eukaryotic cells.

Type of Biomaterial	Loading Regime	In Vitro Cellular Model	Effect on Eukaryotic Cells	Ref.
Porous PVDF and PVDF-TrFE membranes	Static	MC3T3-E1 cell line mouse myoblast cell line (C2C12)	Enhanced cell proliferation	[110]
PVDF film on Ti substrate	Static	BMDSCs	Enhanced cell proliferation and osteogenic differentiation	[111]
PVDF film with Ti layer	Dynamic (mechanical stimulation in a bioreactor)	MC3T3-E1 cell line	Enhanced cell proliferation	[112]
PVDF film	Dynamic (mechanical stimulation in a bioreactor)	Human ADSCs	Enhanced osteogenic differentiation	[113]
Porous HA scaffold	Static	MC3T3-E1 cell line	Enhanced proliferation and matrix mineralization	[114]
HA disc	Static	MC3T3-E1 cell line	Increased cell adhesion, proliferation, and metabolic activity	[115]
KNN ceramics	Static	MC3T3-E1 cell line	Enhanced cell proliferation	[105]
HA/BaTiO_3_ composite	Dynamic (mechanical stimulation with loading device: 3 Hz and 60 N)	Osteoblasts	Enhanced osteoblast growth and bone-inducing activity	[107]
HA/BaTiO_3_ composite	Dynamic (electrical stimulation)	Primary culture of human osteoblasts	Enhanced cell proliferation	[116]
HA/BaTiO_3_ composite	Static	MG-63 cell line (osteosarcoma)	Improved cell adhesion, proliferation, and osteogenic differentiation	[117]
HA biomaterial with KNN layers	Static	Saos-2 cell line (osteosarcoma)	Enhanced cell proliferation	[118]

## Abbreviations

ADSCs	Adipose tissue-derived mesenchymal stem cells
bALP	Bone alkaline phosphatase
BG	Bioactive glass
BMDSCs	Bone marrow-derived mesenchymal stem cells
BMPs	Bone Morphogenetic Proteins
CPC	Calcium phosphate cement
DPSCs	Dental pulp stem cells
E	Young’s modulus
ECM	Extracellular matrix
GAG	Glycosaminoglycan
HA	Hydroxyapatite
HUVECs	Human umbilical vein endothelial cells
KNN	Potassium sodium niobate
LIPUS	Low-intensity pulsed ultrasound
MNPs	Magnetic nanoparticles
MSCs	Mesenchymal stem cells
NF-AT	Nuclear Factor of Activated Cells
NPs	Nanoparticles
PCL	Poly(3-caprolactone)
PE	Polyethylene
PEG	Poly(ethylene glycol)
PIII	Plasma immersion ion implantation
PLA	Polylactic acid
PLCL	Poly(l-lactide-co-caprolactone)
PU	Polyurethane
PVDF	Poly (vinylidene fluoride)
PVDF-TrFE	Poly (vinylidene fluoride-trifluoroethylene)
TCP	Tricalcium phosphate
